# Association of Circulating Follicular Helper T Cells and Serum CXCL13 With Neuromyelitis Optica Spectrum Disorders

**DOI:** 10.3389/fimmu.2021.677190

**Published:** 2021-07-14

**Authors:** Xiaoyan Yang, Jing Peng, Xiaoxi Huang, Peidong Liu, Juan Li, Jiali Pan, Zhihua Wei, Ju Liu, Min Chen, Hongbo Liu

**Affiliations:** ^1^ Department of Neurology, The First Affiliated Hospital of Zhengzhou University, Zhengzhou, China; ^2^ Department of Neurology, Renji Hospital, School of Medicine, Shanghai Jiaotong University, Shanghai, China; ^3^ Department of Neurosurgery, First Affiliated Hospital of Zhengzhou University, Zhengzhou, China

**Keywords:** myelitis lesions, AQP4 antibody, CXCL13, follicular helper T cells, neuromyelitis optica spectrum disorder

## Abstract

**Background:**

Neuromyelitis optica spectrum disorders (NMOSDs) are severe inflammatory diseases mediated mainly by humoral and cellular immunity. Circulating follicular helper T (Tfh) cells are thought to be involved in the pathogenesis of NMOSD, and serum C-X-C motif ligand 13 (CXCL13) levels reflect the effects of Tfh cells on B-cell-mediated humoral immunity. Immune cell and cytokine changes during the dynamic relapsing and remitting processes in NMOSD require further exploration.

**Patients and methods:**

Blood samples were collected from 36 patients in acute and recovery phases of NMOSD, 20 patients with other noninflammatory neurological diseases (ONND) and 20 age- and sex-matched healthy volunteers. CD4+CXCR5+PD-1+ Tfh cells were detected by flow cytometry, and serum CXCL13 levels were assessed by enzyme-linked immunosorbent assay (ELISA).

**Results:**

The percentage of CD4+CXCR5+PD-1+ Tfh cells was significantly higher during the acute phase than during the recovery phase, and serum CXCL13 levels were significantly higher in patients in the acute and recovery phases of NMOSD than in the ONND and control groups. The Tfh cell percentage was positively correlated with CXCL13 levels, and both were positively correlated with Expanded Disability Status Scale (EDSS) scores and cerebrospinal fluid protein levels in patients with acute NMOSD.

**Conclusion:**

Circulating Tfh cells level has the potential to be a biomarker of disease severity.

## Introduction

Neuromyelitis optica spectrum disorder (NMOSD) was originally thought to be a subtype of multiple sclerosis (MS), from which it was not differentiated until Lennon discovered blood aqp-4 antibodies in 2004 (1). NMOSD is more prevalent in areas with non-Caucasian populations, especially in Asian countries (2, 3). While its pathogenesis is not completely clear and requires further research, studies have demonstrated that T cells and many cytokines are involved in NMOSD. Follicular helper T (Tfh) cells are a new subset of CD4^+^ T cells that localize in germinal centres (GCs), show growth and development patterns that are closely related to B cells, and play an important role in directing B cells to secondary lymphoid tissue through interactions mediated *via* a variety of surface receptors and cytokines. Therefore, Tfh cells are also considered an effector T cell type that plays an important role in B cell activation and differentiation. When deregulated, Tfh cells exacerbate humoral responses and the autoantibody production in autoimmune diseases (4). Studies have shown that Tfh cells are CD4+CXCR5+PD-1+, which can be used to quantify them in peripheral blood. C-X-C motif ligand 13 (CXCL13), also known as B lymphocyte chemoattractant-1 (BLC-1), is an important chemokine that directs the migration of B cells before they differentiate into antibody-secreting cells and is mainly expressed by follicular dendritic cells and GC-Tfh cells (5, 6). CXCL13 interacts with CXCR5 (expressed by Tfh and B cells), which promotes B-cell development within secondary lymphoid tissues *via* chemotactic recruitment. Tfh cells are the main source of CXCL13, and serum CXCL13 levels therefore indicate the role of Tfh cells in B-cell-mediated humoral immunity (7). CXCL13 has been shown to have potential utility as a plasma biomarker of GC activity in humans (8). Although Tfh cells and CXCL13 have been studied in various autoimmune diseases, the changes that occur in cells and cytokines during the dynamic relapse and remitting processes observed in NMOSD and their associations with the prognosis and other clinical parameters of this condition still need further exploration. Therefore, we detected the proportion of CD4+CXCR5+PD-1+Tfh cells in the peripheral blood and serum CXCL13 levels in patients with NMOSD and analysed the relationships between both Tfh cells and CXCL13 levels in patients with different stages, subtypes and clinical features of NMOSD. We also explored the correlation between Tfh cells and CXCL13 levels and discuss the roles of circulating Tfh cells and serum CXCL13 levels in the pathogenesis of NMOSD, thus providing new immunotherapy targets for this disease.

## Materials and Methods

### Subjects

Thirty-six neuromyelitis optica spectrum disorder (NMOSD) patients were recruited from the Department of Neurology at the First Affiliated Hospital of Zhengzhou University, China, between October 2016 and December 2017, including 25 relapsing patients and 11 patients with first episode. The inclusion criteria for acute phase patients as follows: 1. Conformed to the 2015 Wingerchuk criteria (9); 2. Serum AQP4 antibody positive; 3. New neurological symptoms and signs or aggravation of the original symptoms lasting for more than 24 hours, and corresponding new lesions were observed on magnetic resonance imaging (MRI). The exclusion criteria for acute phase were previous corticosteroid treatment within 1 month before first sampling, another active immune disorder, infection or cancer. In order to avoid effects of oral immunosuppressants on relapsing patients, we included the relapsing patients who had quit previous maintenance treatment at least 1 month by themselves for side effects, economic burden, pregnancy decision or other reasons, which is far from rare in China. Patients in acute phase were given a venous infusion of methylprednisolone (MPDN) (1000 mg/day) for the first 3 days and gradually decreased to maintenance dosage prednisolone (PDN) which was orally administered. All patients showed an effective response to the MPDN. And then patients were prescribed mycophenolate mofetil as their immunosuppressive agent with starting and maintenance doses of 1000 mg/day. Clinical remission was defined as both neurological symptoms and neurological examination signs that remaining stable for at least 30 days.

The other noninflammatory neurological disease(ONND) group comprised 8 patients with transient ischaemic attack (TIA), 5 with benign paroxysmal positional vertigo (BPPV), 4 with idiopathic epilepsy and 3 with multisystem atrophy (MSA). Twenty age- and sex-matched healthy volunteers without organic diseases were recruited as the healthy control (HC) group. The experimental protocol was approved by the Ethics Committee of the First Affiliated Hospital of Zhengzhou University. The methods were compliant with the ethical guidelines for medical and health research involving human subjects as established by the National Institutes of Health and the Committee on Human Research at the First Affiliated Hospital of Zhengzhou University. All participants provided written informed consent prior to participation. The demographic and clinical characteristics of the patients in each group are shown in **Table 1**.

**Table 1:** Demographic and clinical characteristics of patients in the NMOSD, ONND and HC groups.

Characteristics	NMOSD	ONND	HC	statistics	P
n	36	20	20		
Age	39.95 ± 14.27	42.35 ± 15.78	40.80 ± 14.93	F=0.059	0.942
Female/male	27/9	11/9	15/5	χ2 = 2.793	0.247
Disease duration	4.7 (0,7.6)		–	–	–
Clinical presentation					
Isolate MY, n (%)	18, (50.0)				
Isolate ON, n (%)	4, (11.1)				
ON+MY, n (%)	7, (19.4)				
APS, n (%)	3, (8.3)				
Brainstem syndrome, n (%)	2, (5.6)				
APS+MY, n (%)	2, (5.6)				
CSF protein (mg/L)	420.39 ± 159.51	–	–	–	–
CSF WBC count (10^6/L)	4 (2,6)	–	–	–	–
EDSS score(acute phase)	7.78 ± 1.27	–	–	–	–
EDSS score(recovery phase)	5.93 ± 1.78				

Data are shown as medians and ranges for CSF WBC counts, disease duration and as mean and standard deviations for age, EDSS scores and CSF protein levels.; MY, myelitis; ON, optic neuritis; APS, area postrema syndrome; EDSS, Expanded Disability Status Scale; CSF, cerebrospinal fluid; Normal values, CSF WBC count, 0–8 × 10^6 cells/L; CSF protein concentration, 150–450 mg/L; disease duration,years; *P* < 0.05 versus data for HCs.

### Data and Sample Collection

Demographic and clinical features, cerebrospinal fluid (CSF) parameters, Expanded Disability Status Scale (EDSS) scores, laboratory and imaging examinations and therapy data were collected in the acute phase. To elucidate the dynamic changes in Tfh cells and CXCL13 levels in different disease stages, venous blood samples were collected before treatment and four to eight weeks after MPDH treatment. Each blood sample was centrifuged within 1 hour, and the serum supernatant was collected and stored in Eppendorf centrifuge tubes at -80°C until use. Anticoagulant blood samples were detected within 6 hours. White blood cell (WBC) counts, protein levels, and immunoglobulin G (IgG) levels in the CSF were routinely examined in the hospital.

### Flow Cytometric Analysis (FCM)

Tfh cells were defined as CD4+CXCR5+PD-1+ cells. Anticoagulant blood was incubated with fluorescein isothiocyanate (FITC)-anti-CD4, allophycocyanin (APC)-anti-CXCR5, phycoerythrin (PE)-anti-PD-1 mouse anti-human monoclonal antibodies (mAbs) or appropriate IgG isotype controls (BioLegend, USA); in the blank control group, no reagents were added; All experiments were performed in triplicate at room temperature in the dark for 30 minutes, and the reactions were then treated with lysing solution and washed with phosphate-buffered saline (PBS). The cells were detected by flow cytometric analysis using a BD FACSCanto II (BD Biosciences, USA). The data were then analysed with FlowJo software (version V10).

### Measurement of CXCL13 Concentrations

CXCL13 concentrations were measured using a commercially available (BLC-1/CXCL13) enzyme-linked immunosorbent assay (ELISA) kit (R&D Systems,USA). The absorbance was measured at 450 nm, and serum concentrations of CXCL13 were calculated according to standard curves.

### Statistical Analyses

For data that met the assumptions of normality (Shapiro-Wilk W tests), the results are expressed as the mean ± standard deviation; otherwise, the results are expressed as the median (P25, P75). Differences between two groups were compared using a t test or the Wilcoxon test according to normality. Comparisons of variables among three groups were analysed by analysis of variance (ANOVA) (for normally distributed data) or the Kruskal-Wallis test (for non-normally distributed data) as appropriate. Differences among qualitative variables were assessed with Pearson’s chi-squared test. The Bonferroni method was used for pairwise comparisons of differences between groups. Correlations were analysed using Spearman’s correlation coefficient. A two-sided P-value of < 0.05 was considered statistically significant. Statistical analyses were performed using SPSS software (v. 21.0). All figures were constructed using GraphPad Prism software (v. 5.0).

## Results

### Clinical Features of Individuals

The demographic and clinical features of individuals are shown in **Table 1**. There were no significant differences in the sex or age distributions among the NMOSD, ONND and HC groups.

### Proportion of Tfh Cells and Serum CXCL13 Levels in Peripheral Blood in the Three Groups

**Figure 1:**
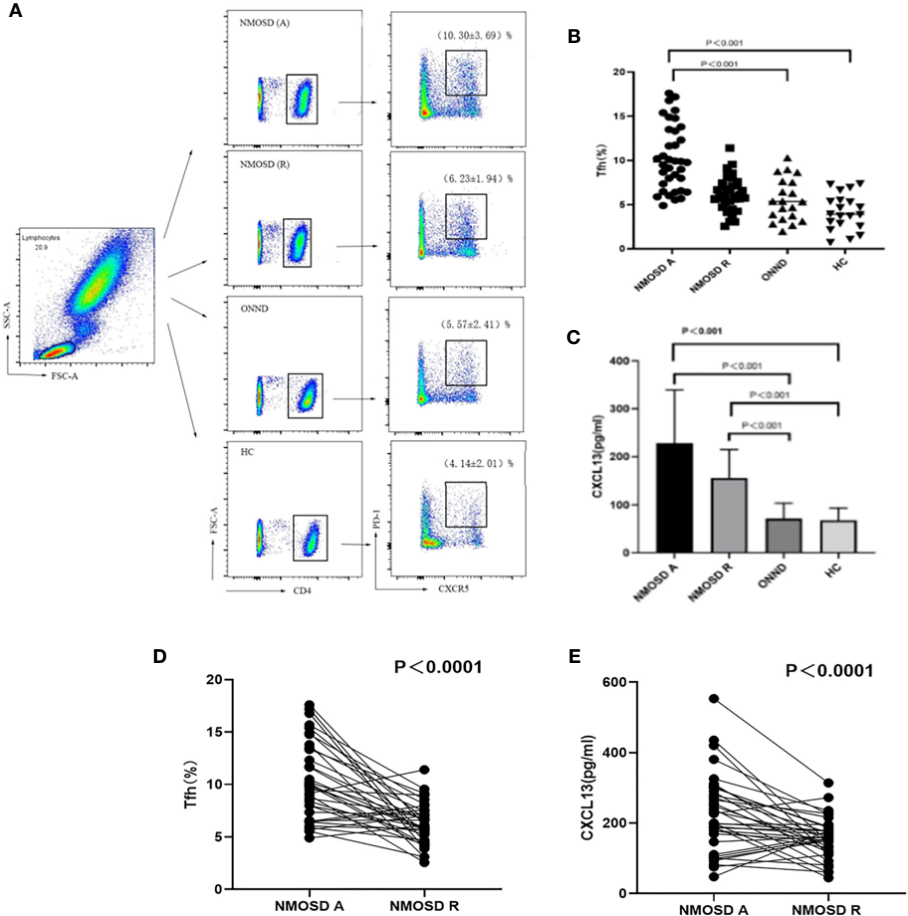
FACS analysis of circulating Tfh cells in individual participants. The cells were gated to obtain results for lymphocytes, CD4+, CXCR5+ and PD-1+cells. **(A)** Flow cytometric analysis **(B, C)** Circulating Tfh cell ratio and serum CXCL13 levels in the acute and recovery stage of NMOSD, ONND and HC groups. **(D)** Comparison of circulation Tfh cell ratio in NMOSD. **(E)** Comparison of serum CXCL13 level in NMOSD. Each line represents the changes of Tfh and levels of CXCL13 in relapsing and remitting stage of NMOSD patients. NMOSD A, acute phase of NMOSD; NMOSD R, recovery phase of NMOSD).

When all groups were compared, we found that the proportion of CD4+CXCR5+PD-1+ Tfh cells was higher in the acute NMOSD group than in the recovery group, ONND group and HC group (P < 0.01, P < 0.001, P < 0.001; **Figures 1A, B, D**). The statistical method for analysis of CXCL13 levels was the same as that used for Tfh cells. There were significant differences among the three groups in serum CXCL13 levels, which were significantly higher in patients in the acute (227.67 ± 111.22 pg/ml) and recovery (156.35 ± 58.40 pg/ml) stages of NMOSD than in the ONND (71.80 ± 31.97 pg/ml) and HC (46.10 ± 24.48 pg/ml) groups (P < 0.001, P < 0.001; P < 0.01, P < 0.001; **Figures 1C, E**).

### Correlations Among the Proportion of Circulating Tfh Cells, CXCL13 Levels, EDSS Score, CSF Protein Levels and Cell Numbers in Patients With NMOSD

**Figure 2:**
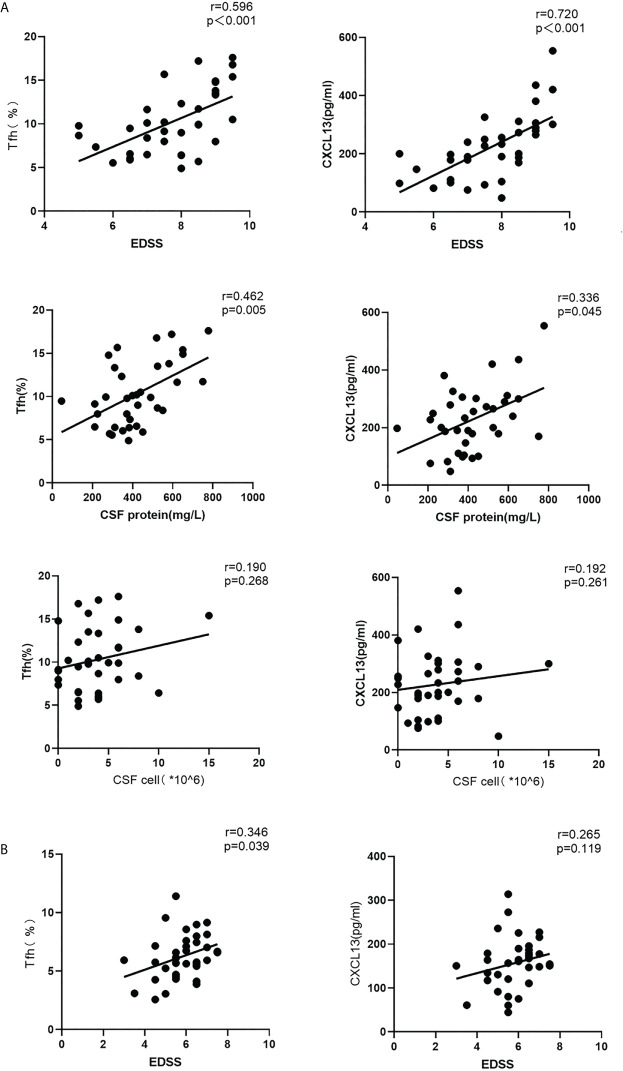
Correlations among the circulating Tfh cell ratio, CXCL13 levels, EDSS scores, CSF protein levels and CSF cell numbers in the acute and recovery stages of NMOSD **(A)** acute stage; **(B)** recovery stage. r: correlation coefficient.

In acute stage NMOSD patients, both the proportion of Tfh cells in the peripheral blood and serum CXCL13 levels were positively correlated with EDSS scores (r = 0.596, P < 0.001; r = 0.720, P < 0.001), and CSF protein content (r = 0.462, P <0.01; r = 0.336, P = 0.045), but there was no obvious relationship between the cell number in the CSF and either the proportion of Tfh cells or serum CXCL13 levels (r = 0.190, P = 0.268; r = 0.192, P = 0.261) (**Figure 2**), A positive correlation was found between the Tfh cell ratio and EDSS scores in the recovery stage (r = 0.346, P = 0.039), but there was no significant relationship between CXCL13 levels and the EDSS score (r = 0.265, P = 0.119) during this stage (**Figure 2**).

**Figure 3:**
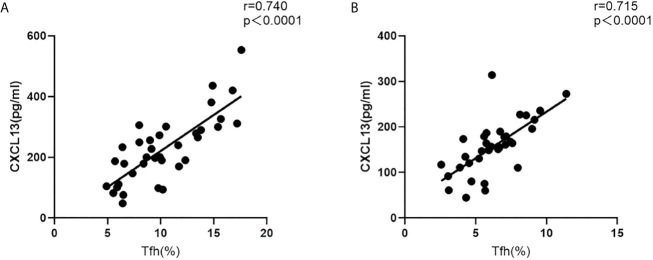
Correlation between the circulating Tfh cell ratio and serum CXCL13 levels in NMOSD patients. **(A)** acute stage; **(B)** recovery stage (r: correlation coefficient).

Furthermore, in both the acute and recovery stages of NMOSD, the circulating Tfh cell ratio and serum CXCL13 levels were significantly and positively correlated (r = 0.740, P < 0.001; r = 0.715, P < 0.001) (**Figure 3**).

### Relationship Among the Proportion of Tfh Cells in Peripheral Blood, Serum CXCL13 Levels, and Myelopathy in Patients With NMOSD

**Figure 4:**
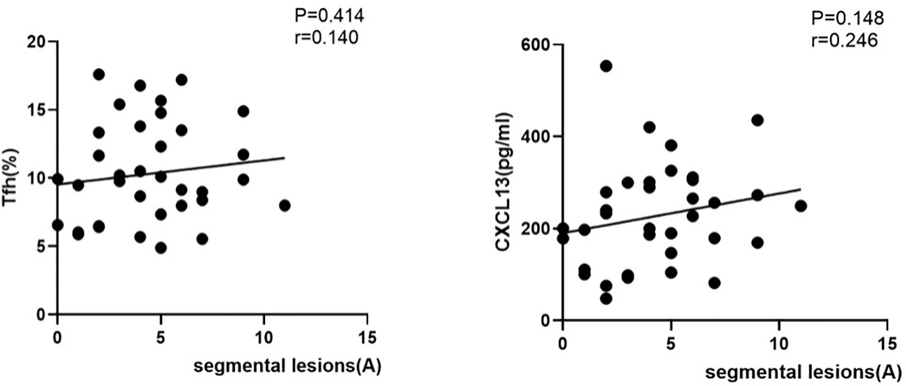
Correlations among the proportion of circulating Tfh cells, serum CXCL13 levels, and segmental lesions in patients with NMOSD. (Correlation among the proportion of circulating Tfh cells, serum CXCL13 levels and segmental lesions in NMOSD patients with positive serum antibodies. A, acute phase of NMOSD; r: correlation coefficient.

In spinal cervical NMOSD patients with positive serum AQP4 antibodies, there was no obvious relationship between segmental lesions and either Tfh cells or serum CXCL13 levels during the acute stage of NMOSD (r=0.140^#^, P=0.414; r=0.246^#^, P=0.148; **Figure 4**)

## Discussion

Tfh cells are important for the humoral immune response, especially for the production of pathogenic antibodies. Tfh cells can be produced in GC and lymphatic tissues, enter the blood circulation, and exist in peripheral blood in various forms. In addition, Tfh cells have been reported to be involved in the pathogenesis of a variety of autoimmune diseases, such as MS (10), myasthenia gravis (11), and systemic lupus erythematosus (12). However, there is currently no study that definitively shows the causal effects of Tfh cells in NMOSD.

We studied the Tfh cell ratios in peripheral blood and the changes in CXCL13 levels at different stages of NMOSD patients, and found that the proportion of Tfh cells and serum CXCL13 levels in the peripheral blood were significantly elevated in the in acute-phase group which was positive for serum AQP4 antibodies. These increases were associated with acute disease activity, including EDSS scores, CSF protein, etc., and is consistent with past research results (9, 13–16). This implies that the Tfh ratio and CXCL13 level were related to the pathogenesis of NMOSD. We further found that both CXCL13 and Tfh were positively correlated, suggesting an interaction and relationship between the two in the incidence and outcome of NMOSD. Tfh cells expressing CXCR5 are necessary for the final differentiation of plasma cells in germinal centre. CXCL13 can bind to the CXCR5+ receptor located on the surface of Tfh cells and recruit Tfh and B cells into the GC, which contributes to the contact between Tfh cells and B cells in GC to produce pathogenic antibodies (17, 18). Interestingly, GC-Tfh cells can also produce IL-21 and CXCL13, which bind to the IL-21 receptor and CXCR5 on Tfh cells, respectively, to induce differentiation and recruitment of Tfh cells. We speculated that the activation of the autocrine loop causes excessive expansion of Tfh cells, leading to overreaction of the germinal centre to cause excessive production of high-affinity pathogenic immune antibodies. Prior studies have confirmed that Tfh cells and related molecular disorders may participate in regulation of NMOSD onset. Fan and others found that in patients with NMO/NMOSD, CCR7-ICOS+ memory Tfh cells were increased in the peripheral blood and CSFand that IL-21 in the peripheral blood and CSF of these patients was closely related to the function of Tfh cells. Memory Tfh cells and IL-21 also showed a positive correlation in these diseases (14). It has also been noted that Tfh cell subsets are present in disproportional amounts in patients with NMOSD, which may lead to slow B-cell activation and sustained antibody production. The application of B-cell deletion therapy, i.e., rituximab can significantly reduce circulating Tfh ratios and restore Tfh subsets (19, 20). In addition, some studies found that defects in the surface molecule PD-1 led to an increase in Tfh cells and a decrease in the expression of IL-4 and IL-21, thus resulting in a decrease in long-lived plasma cells (15).

When the disease entered the stable remission stage, we found that frequency of Tfh cells was significant decrease after effective corticosteroid treatment,which was consistent with the results of previous studies (9, 15, 16, 21). Corticosteroids were reported to improve the Tfh cell immune response by reducing the serum glucocorticoid-regulated kinase 1(SGK1). It is reported that monocytes and follicular dendritic cells can produce CXCL13 and may increase plasma CXCL13 concentrations in different inflammatory environments (22, 23). However, GC-Tfh cells still are an important,perhaps a major, producer of CXCL13. Colin showed a strong correlation between CXCL13 and lymphoid tissue resident GC-Tfh cells and monocyte-generated CXCL13 may not significantly impact plasma CXCL13 levels, therefore, plasma CXCL13 can act as a plasma biomarker for GC activity in generally inaccessible lymphoid tissue (8). Thus, we speculate that abnormal CXCL13 elevation may indicate remission in NMOSD patients with high GC activity and activation of the immune system. This mechanism may be associated with recurrence of the disease. Previous studies have found that CXCL13 levels tended to be high in the serum and CSF of patients with recurrent NMOSD and MS, although CXCL13 level was higher in NMOSD patients than in MS patients. The level of CXCL13 in the CSF was correlated with EDSS scores, ARR, and the number of CSF cells. Serum CXCL13 level was correlated with the degree of remission and changes on MRI (21). In addition, Peng-Peng IP et al. suggested that the elevation of serum CXCL13 in patients with NMOSD in the remission phase might indicate the presence of CXCL13 in their central nervous system (23). Given that blood samples are easily available and that CXCL13 in both the central nervous system and blood may reflect immune activation in the central nervous system, routine monitoring of peripheral blood CXCL13 levels in patients with NMOSD is feasible for understanding and evaluating disease progression. Other studies have suggested that co-determination of serum CXCL13 has potential in allocating the focus of the inflammatory process, which may localize to the periphery or to the CNS or co-localize in both compartments with possible therapeutic consequences (24). These results are consistent with our findings. We speculate that CXCL13 has the potential to be a new target for the treatment of NMOSD, and the CXCL13 neutralizing antibody MAB 5261 or other drugs may have potential for NMOSD treatment (25).

This study has the following limitations The number of patients included in this study was relatively small. Second,lumbar puncture is not routinely recommended for NMOSD in the remission period in China, therefore, it is difficult to obtain CSF-related data during this period. All these important limitations should be addressed in subsequent prospective studies. However, our results further confirm that both humoral immunity and cellular immunity are involved in the pathogenesis of NMOSD, which may provide a unique new target for the clinical diagnosis and treatment of this disease. In addition, Tfh cells secrete a variety of cytokines, such as IL-21 and IL-4, and these findings need to be further expanded and studied to provide a research basis for the pathogenesis of NMOSD.

## Conclusion

In conclusion, the present research demonstrated the alteration of circulating Tfh and CXCL13 in different disease stages. We also described the relationship between Tfh cells and disease activity. The factors evaluated in our study were limited, further studies are required to identify the underlying mechanisms governing the levels of Tfh cells and CXCL13 in NMOSD.

## Data Availability Statement

The raw data supporting the conclusions of this article will be made available by the authors, without undue reservation.

## Ethics Statement

The studies involving human participants were reviewed and approved by the Ethical Committee of the First Affiliated Hospital of Zhengzhou University. Written informed consent to participate in this study was provided by the participants’ legal guardian/next of kin. Written informed consent was obtained from the individual(s), and minor(s)’ legal guardian/next of kin, for the publication of any potentially identifiable images or data included in this article.

## Author Contributions

XY, JP, and HL drafted the manuscript. PL and HL designed the research project. PL prepared the figures. JLP and ZW analysed the data. JLi performed the experiments. JLiu and MC edited and revised manuscript. All authors contributed to the article and approved the submitted version.

## Funding

This work was supported by Henan Provincial and Ministerial Co-construction Projects (No. SB201901018) and the National Natural Science Foundation of China (No.U2004128).

## Conflict of Interest

The authors declare that the research was conducted in the absence of any commercial or financial relationships that could be construed as a potential conflict of interest.
